# Supplementary material for: Prevalence and associated factors of needle stick and sharps injuries among healthcare workers in northwestern Ethiopia
Source: PLoS One. 2021 Sep 24;16(9):e0252039. doi: 10.1371/journal.pone.0252039 (PMC8462737; doi:10.1371/journal.pone.0252039)
Supplement: Attachment — Submitted filename: Final-Response to reviwers.docx [file pone.0252039.s002.docx]

**Response to the rejected paper of PONE-D-20-044818, PONE-D-16729 and PONE-D-20-30423**

**Response to reviewers for the rejected paper and the paper re-submitted as a new submission for the second time.**

**For the rejected paper PONE-D-20-04818**

Reviewer #1: The topic is very important and certainly of interest. However, numbers of similar studies were done in Ethiopia including the study area including systematic review and meta-analysis. So it will add nothing rather than redundant reports.

***Response: Yes, but there is no any study in the study area so far, thus this current study will help to take timely information in the study area and similar settings (please see the revised version for that explanation of our gaps of the study in lines 79 to 97 in the introduction).***

Reviewer #2: please see all the comments which were written in the PDF format and comment section. Please revise the method sections (partly) the result (descriptive) must be presented in separate section than the analysis table recommendation focus on the conclusion and the data you have

***Response: All comments within the PDF are addressed very well (Please see the revised methods section in lines 120 to 143 and result version in lines 266 to 283 and Tables 1-6)***

- ***Check the revised study area: Lines 107 to 111***
- ***Check the revised sample size: Lines 120 to 128***
- ***Operational definitions: Lines 161 to 166***
- ***Data management and analysis: Lines 199 to 211***

Reviewer #3: this manuscript is not technically sound but there is grammatical problems throughout the document and the data even if the conclusions are based on the finding. as a result An overall proofread is certainly required. Data collection procedures and extraction could be explained in more detail and properly. In addition the address of the authors is not fully completed, even the PI is not known that indicates write up problems and poor manuscript preparation. Line 42 sleep disturbance is reported as a significant factor which is difficult to measure sleep disturbance among health care workers that might have nearly similar pattern of sleep disturbance due to the similar nature of the profession. So how can assess sleep disturbance across and within the professionals (e.g. all nurses might do for similar tasks over night for equal time based on thier night duty shifts). Hopefully all of them might have such problem as far as they are in the same environment. So what do you recommend for this variable?.

***Response: The manuscript preparation is highly modified and updated based on the journal guidelines. Any errors were addressed as suggested (see all sections of the revised version).***

5. Line 40 nurses are more risky than other professionals. Then what do you recommended for this? Did you recommend not being a nurse?

***Response: To reduce risk for nurses, using proper personal equipment and rotation of working environment is good. But it does not mean Nurse should stop working (See lines 314 to 320 in the discussion)***

6.Line 40 (AOR = 3.65, 95% CI [1.08-12.26], P-value) better to re- write as AOR (95%CI,3.65(1.08-12.26) throughout the document.

***Response: It is done as recommended. Thank you and sorry for creating such minor error.***

7. Line 42 sleep disturbance is reported as a significant factor. How do you measure sleep disturbance? All of your respondents are health workers that might have nearly similar pattern of sleep disturbance due to the nature of the profession. So how can assess sleep disturbance across and within the professionals (eg. all nurses might do for similar tasks over night for equal time). Hopefully all of them might have such problem as far as they are in the same environment. So what do you recommend for this variable?

***Response: Since the study is cross-sectional, data is measured by self-reporting and health professional who feel sleepy during the working time is considered as sleep disturbance.***
8. Line 44 you concluded that the proportion of NSSI is slightly high. Do,t you think that 29.5% is not high?

***Response: Compared to the national coverage it is not high but compared to its magnitude is high. SO our comparison is with Ethiopia coverage of need stick injuries, which is around 50% in 2016.***

Introduction
9. The narration section of this part of the manuscript could be improved. It is not easy to follow the idea.

***Response: This concern is addressed well. Please see the revised version lines 57 to 64.***

10. Line 56 better to use Vancouver referencing style throughout your document instead of Harvard style.

**Response: Vancouver is used and please see the revised version in each section.**

11. Line 58-64 not well synthesized rather simply putted what the science says. No need of writing the science here rather better to wrote the evidence.

***Response: This concern is revised. Please see the revised version 66-72.***

12. Line 66-70 needs re –phrasing

***Response: it is done (See lines 74 to 77).***

13. Line 70 use of oldest reference (2005) is not recommended. If it is possible it is better to use references 5 years back if not we can use up to 10 years. But using such oldest reference is not recommended.

***Response: At-least 10 years of reference used.***

14. Line 70 is it sounded that the knowledge of HCWs is too low in order to being exposed for NSSIs?

***Response: yes, the data shows it is low.***

15. Line 74-76 not appropriate for introduction. It seems literature review.

***Response: It is deleted. Thank you for the comments.***

16. The flow of ideas could be from global to local. Eg. line 71 about Amhara region,line 77 about Africa and line 82 about Ethiopia.

***Response: This concern is addressed well. Please see the revised version in lines 74 to 97.***

17. Line 82-83 you reported that poor documentation contributs for the high proportion of NSSIs. How it can be?

***Response: This concern is addressed well. Please see the revised version.***

18. Line 85-87 reported that no more studies are conducted in Ethiopia but there are a numbers of studies in Ethiopia( eg awi zone, tigray region, bale zone, Bahir dar and so on.. even there are 431 literatures’ done in Ethiopia as one systematic review study reported. So what makes your finding unique??

***Response: There was no similar study in the study area and our study gives current evidence, however previous studies in Ethiopia are almost old and not in the study area (See the revisions in 97 to 97).***

Methods and materials section better to be edited as methods

***Response: all the methods and material section is edited carefully. All These concern below are addressed well. Please see the revised version in lines 99 to 222.***

20. Line 96 better to be written as the study was conducted in south Gondar zone public hospitals

***Response: It is re-written as suggested (see lines in 100 to 102.***

21. Line 98-99 how many zones are there in your study?

***Response: There are only 18 woredas and one zone (see lines 107).***

22. Line 102-103 do you mean governmental hospitals?

***Response: yes, in Ethiopia, public hospital means government hospitals.***

23. Line 104-108 not important rather better to show which districts were selected for the study and how? Similarly which health organizations were selected and how were selected?

***Response: Thank you for the comments and please see the selection of hospitals in 130 to 144.***

24. Your methodology section lacks to show the paired, design and number of respondents.

***Response: Please see the revised version in lines 130 to 144.***

25. Line 114 why you assess your participants for 12 months back? How do you see the recall bias?

***Response: Although there is recall bias, but is it’s a standard as WHO is recommended (see lines 151 to 154).***

26. Line 115-117 better to list out in number for each profession.

***Response: It is listed out, see the source population in lines 115 to 118 and also see in Table 1.***

27. Line 120-124 better to revise by including the formula and how it was calculated.

***Response: revised as suggested and see lines 120 to 128.***

28. Line 124 you got 306 but it says 295 at the abstract section.

***Response: sorry for this error. It is corrected as 306 as sample size and 296 as the final size of data collected (See lines 120 to 128).***

29. Line 128 does internists are not considered as medical doctors?

***Response: Internist are medical doctors but they are specialized***

30. It is better to merge the sample size determination with the sampling procedure by omitting the operational definition in between.

***Response: It is merged and see lines 119 to 144.***

31. Line 158 what mean by the word created? Do you mean adapted?

***Response: This is error and deleted***

32. Line 163 for whom the 3 nurses were assigned to facilitate? Are they data collectors?

***Response: yes, they are data collectors (see lines 174 to 175).***

33. Line 170 where did the pre-test done?

***Response: In another hospitals that are not part of the study area and see in lines 185 to 187.***

34. Line 175 data entry could be part of data analysis

***Response: ok, it is revised as suggested (see lines 197).***

35. Line 184 AOR shows the measure of association not the significance level

**Response: Agreed**, Thank you for the comments.

36. Could you submit the ethical letter you got from ERC of Wollo University Result

**Response; Yes**

37. Line 205 better to talk about respondents not about questioners

**Response: Ok, done (See lines 167 to 178).**

38. Line 204 better to talk about 295 respondents that responds fully for your questioner

***Response: ok***

39. Line 204-212 better to re-write again

***Response: re-written***

40. Line 210 how many respondents from each profession?

***Response: Please see Table 1***

41. How do you appreciate the difference among different profesionals? Don’t you do anova /students t- test?

**Response: Since our study has not have continues outcome, no need ANOVA for our data analysis methods (Please see the revised data analysis 197 to 211).**

42. Poor manuscript preparation
⎫ All tables should be below the reference
⎫ Better if table 1 is about socio-demographic characteristics
⎫ Some narration is needed for each table eg. Table1

***Response: This concern is addressed well. Please see the revised version for all sections.***

43. On table 3 there is a variable ” working night shift” but I don’t think wither it is variable across the professionals as well as between the professions because it is common for all health workers and the same is true for universal guidelines, safety protocol, infection prevention committee, place of sharp material, number of working hours…..so on

***Response: This concern is addressed well. Please see the revised version.***

44. Table 4
⎫ There is a variable did you report NSSIs, then 21 individuals reasoned NO.
⎫ But on the 2nd question “ to whom did you reasoned” all 87 respondents give answer for this question. But it should be 66(87-21). How it could be? Again the third question “why did not report” in the same way all 87 gives response. How it can be??

***Response: It is error and corrected. This concern is addressed well. Please see the revised version.***

45. Line 295-299 which one is your reference/ baseline?

***Response: not showed clearly***

46. Table 6
47. You put the educational label as diploma, below diploma, medical doctor, MSc….. so on. How it can be? Do you got any health professional below diploma?

***Response:*** We got health professional below diploma*.* This is because education status of lower indicates that there are healthcare workers who have not diploma such as cleaners and laundry staff of the hospitals (See footnote of Table 1 and Table 6).

**Discussion**
Needs extensive revision with scientific justification for the discrepancies b/n yours and other studies
Line328-329 unclear no need of justification for similarities

***Response: This concern is addressed well. Please see the revised version.***

**Conclusion**

Its needs revision for write-up, but throughout.

***Response: It is done in a high careful manner,***

**Limitations**

No limitations and strengths are explained

***Response: This concern is addressed well. Please see the revised version.***

48. Line 402-404 list of abbreviations better to be abbreviations and better to be write separately. Not in paragraph form

***Response: This concern is addressed well. Please see the revised version.***

49. This study lacks Declarations, regarding to
⎫ Ethics approval and consent to participate,
⎫ Consent for publication,
⎫ Availability of data and materials,
⎫ Competing of interests,
⎫ Funding sources
⎫ Author’s contribution, and affiliation,
⎫ Weather they have publication during the past 5 years
⎫ recent grants
⎫ financial relationship with the company who funded the research if there an this paper needs major revision

***Response: This concern is addressed well using the journal guidelines. But the format you suggested is works for BMC journals not for PLOS ONE***

Reviewer #4: 1. Using abbreviations in abstract part is not recommended, rather you can paraphrase ideas to minimize numbers of words.

***Response: This concern is addressed well. Please see the revised version.***

2. You just used a single reference for a large paragraph, but you can synthesize sentences

***Response: This concern is addressed well. Please see the revised version.***

3. There are many health centers in the study area, so why you don’t include them?

***Response: We only studied hospitals, not all health facilities***

4. Why you use correction formula? because you have no a problem of sample in your study area. For me it will reduces representativeness of the study.

***Response: The source population is very small and using correction formula is ok because the calculated sample size becomes nearly equal with all the healthcare workers in the hospitals***

5. You mention that, design effect 2 is used due to multistage sampling, your sample size after correction formula and non-response rate is 306.So 306*design effect 2=612, but your final sample size is 306.For me the result of the study is not representative and logical.

***Response: This is error (please see the sample size calculation in lines 120 to 128.***

6. Your operational definitions seem standard definitions, even your outcome is not well operationalized.

***Response: This concern is addressed well. Please see the revised version.***

7. I am not clear about the instruments used to measure both the outcome and independent variables. It needs strong clarification! It is difficult to measure sleep disturbance by simple question with yes or no, regarding substance use, have you asked ever or current use? What about frequency, amount, type of alcohol. Even the knowledge part, is that possible to assess knowledge with a single yes or no question? How did you control social desirability bias?

***Response: This concern is addressed well. Please see the revised version.***

8. Did you have performed multicollinearity test for variables, information, training and knowledge?

***Response: Yes***

9. In the result part, interpretation for predictors is too long and vague

***Response: This concern is addressed well. Please see the revised version.***

10. In the discussion part, better to put CI for prevalence to readers easily compare with other studies. Explanations for difference in prevalence seems poor, for example paragraph 6.
11. How cross-sectional study design nature limit your study???

***Response: This concern is addressed well. Please see the revised version.***

12. Why you recommend cohort and qualitative studies to be performed?
*this study is over researched in the nation and you do in the same manner with previous ones'. I don't think it will add new findings.

***Response: In order to find out causal factors, cohort study is strong than cross-sectional survey and qualitative study will help to explore the existing barriers for need stick injuries.***

Reviewer #5: 1. How could your sample size is representative of HCWs working in south Gondar hospitals? it is too small!

***Response: The sample size is more than half of the total health workers in the three hospitals. Since the source population is not higher, our sample size is more than enough.***

2. I did not see the sample size calculation for factors. Did you compute sample size for factors?

***Response: It was calculate for factors, but we believe that all the details should not be written in the manuscript. The sample size calculate by prevalence is higher than calculated by factors and then we remove it.***

3. what makes different your research entitled with Needle Stick and Sharps Injuries among Healthcare Workers in South Gondar Zone Hospitals, Northwest, Ethiopia from a paper done at Bahir dar?

***Response: Our study done in 3 hospitals from different place, whereas the Bahir Dar study is in one hospital with old study period (see the study area in lines 100 to 111).***

4. did you mean that you took the prevalence of needle stick and sharp injuries among HCWs working in BDR or you select the largest sample size after you calculate for different researches done in Ethiopia using their prevalence?

***Response: Yes, we calculate the sample size using various prevalence and then we took the maximum sample size.***

Reviewer #6: the publication doesn't understand duplication of research in the nearest study area and better to do in specific professionals. It was not referenced based on submission guideline.it has unnecessary tables and not well structured. Introduction was not logically ordered.

***Response: Since the health professionals in each category are small in number, it is hard to do research for each category. But this idea is fine and will be considered for our next study project.***

**Response to reviewers for the rejected paper PONE-D-20-16729**

Title: Prevalence and associated factors of injuries among Health Professionals in Ethiopia

Dear Carmen Melatti,

Dear Sir, thank you for letter and we feel that the revised version is suitable for PLoS ONE further consideration and we re-submitted as new submission for your attention.

I found that we had a limitation to develop a good response letter that is informative and self-explanatory to readers, however, I addressed all the concerns within the revised version. For your concern, please see our response here below Q1: As we understand the reports, the reviewers and previous Academic Editor raised overlapping concerns on the contents of the manuscript. Specifically, they highlighted several issues with the methodology used (for example, with the validation of the questionnaire and the sampling methods used).

Response: The sampling methods explained in a very detail manner and other researcher can replicate it. Please see lines 130 – 144 in page 7. Furthermore, he data collection tools, data collection and quality assurance methods were explained in lines 168-194 as follows:

Data quality was assured during questionnaire design and data collection, entry, and analysis. The questions were objective, non-leading, logically sequenced, and free of scientific jargon. To ensure the validity of the data collection tool, inter-observer reliability was ensured by providing clear definitions of measured variables, and events to be recorded. We re-self-administered 5% of the study participants to check reliability of the information entered at different times of the same study participant. Furthermore, to ensure the content of the survey tool is valid, the questionnaire was pretested in a 10% sample of the study’s sample of HCWs in nearby hospital (Lay Gayint Hospital). Based on the pre-test responses, questions were revised as necessary.

The principal investigator provided one day’s training to data collectors, and then reviewed the collected data each day, returning incomplete questionnaires to data collectors who in turn contacted study participant the same day. In order to reduce social desirability bias in answers, a self-administered survey and closed-ended questions were used. In order to verify the accuracy of data entries, two generic data verification strategies were employed as described in another study [26]. As the first step, randomly selected 10% of the questionnaires were thoroughly checked. Following this, descriptive statistics, results from cross-tabulations, and frequency distributions were examined before performing statistical analysis.

Q2: Unfortunately, we do not feel that these concerns have been sufficiently addressed; we also note that the response to reviewer provided does not adequately respond to most of the comments, as it does not specifically refer to modifications performed in the text.

Response: We found that I had a limitation to develop a good response letter that is informative and self-explanatory to readers, however, I addressed all the concerns within the revised version.

**Response for the rejected submission PONE-D-20-30423**

**Dear Editor,**

**Table 6 about education status: sorry for the error about our response of diploma and lower education status.** We got health professional below diploma*.* This is because education status of lower indicates that there are healthcare workers who have not diploma such as cleaners and laundry staff of the hospitals.

**Sample size calculation:**

The sample size was determined using a single population proportion ($n=\frac{(z_{a/2})^{2}*p(1-p)}{d^{2}}$) formula [24] with an assumption of Z_α/2_ at 95% confidence interval is 1.96; *d* is degree of error of 5%; and proportion (*p*) of NSSIs among HCWs of 32.8% was taken from a study done in Debre Birhan hospitals in Amhara Region [19]. We used a design effect of 1.5 since we employed a multi-stage sampling method, giving a calculated sample size of 508. Furthermore, since the source population in South Gondar Zone hospitals was less than 10,000, we used a sample size correction formula of *n*/[1+ (*n*-1)/*N*] [24], where *n* is the initial calculated sample size (508) and *N* is the source population (749). Then, the sample size became 303. Finally, a 5% non-response rate was added and a final sample size of 318 was obtained.
