# Supplementary material for: Prevalence and associated factors of needle stick and sharps injuries among healthcare workers in northwestern Ethiopia
Source: PLoS One. 2021 Sep 24;16(9):e0252039. doi: 10.1371/journal.pone.0252039 (PMC8462737; doi:10.1371/journal.pone.0252039)
Supplement: Attachment — Submitted filename: Response to reviwers.docx [file pone.0252039.s004.docx]

**Response to reviewers**

Reviewer #1: Authors revised the manuscript. Some have been addressed, but some modifications still required.
**Response: Thank you for your positive remark for our revision. We revised the manuscript as per your comments and please see the answers herewith.**
1. Line 167: operational definition of universal precaution: Avoiding contact with patients’ bodily fluids. It should be the "the practice in medicine to avoid contact with patients' bodily fluids". Please provide reference as other operational definition.
Response: We cited reference for the definitions of universal precaution (see in page 8 line 182).
Comments: As suggested before, the author need to use more standard definition and cite from appropriate reference. The citied reference is not easy to find and wrongly written.

**Response: We really appreciate your important comment. We updated the definitions and the reference based on your comment. Please see the revised version under the operational definitions in page 8.**

2. Why did you combine internist, ophthalmologist, and other specialist in the same group with health officers who might not have the same chance of exposure to patients' bodily fluid? Why not combine with medical doctor? Please clarify the classification or reclassify as other writers do.
Response: This is because of the frequency of the data. In each hospital the number of internist, ophthalmologist, and other specialist is mainly one or two or three and even sometimes no in the specific category. To make the model suitable during the data analysis, we merged that very less frequent profession in the same category regardless of the exposure level.
Comments: I still found the reason of combining these specialists with health officers not reasonable. Especially, it was just based on suitable data analysis.

**Response: Thank you for your reasonable questions. I have to brief about the role and responsibilities of health officers in Ethiopian healthcare context. Health officers in Ethiopia are doing the same job as physicians where there was no physicians in the hospital. Moreover, even when there is physicians, health officers is do similar job and the various of activities depends on the level or status of the cases (patients) degree of severity. In all these circumstance, the exposure status is similar. Therefore, merging of our data is reasonable.**

3. Table 3: receive HBV prophylaxis. Is it HBV immunoglobulin or HBV vaccination before working in health care institution? Did you consider their knowledge of their own anti HBs level? Please clarify in method section.
Response: Thank you for this important questions. We mean that HBV vaccination before working in health care institution. We noted this in Table 2 foot note and please see Table 2.
Comment: It is better to call as HBV vaccination (completed or not-completed). I assumed the author did not have data on HBV vaccine completion.

**Response: Yes, as you noted very well, the limitation of our data did not consist about the completed and not-completed HBV vaccination. We are sorry for missing such data and hoping that this will not be a major problem for this paper.**

4. Table 4: How do you define participants' knowledge about prevention of NSSI? Please clarify items and scoring used in method section.
Response: Thank you for this key questions. We made error by saying knowledge, but our study was either the know NSSI can be prevented or not, we updated Table 4. We did not study knowledge using different items of questions.
Comment: Does it mean prevention NSSI or prevention of infection caused by NSSI? These are two different terms
**Response: Thank you for your insight. Prevention NSSI and prevention of infection caused by NSSI are two different ideas. However, in our study context, we only studied as NSSI can be prevented or not, whereas we did not study about prevention of infection caused by NSSI.**

5. It is interesting that 8.1% of NSSI happened while walking in the hospital. Can you explain what kind of situation in the study setting that even when walking in the hospital, someone could get NSSI.
Response: Thank you for your concern. Walking in the hospital means that when going to toilet, during moving from one ward to the other ward, during moving from one location to other location during tea break time and etc.
Comment: If this was the situation in that hospital, I would suggest the author describe with more data and emphasize more in the discussion. Please also include realistic suggestion to this horrible situation.

**Response: Many thanks for this valuable comment. We included this in the discussion. About 8.1% of NSSI happened while walking in the hospital. This might be due to while walking in the hospital during visiting toilet, during moving from one ward to the other ward, during moving from one location to other location during tea break time. This also might have been as a result of lack of cleanness of the hospital compound from sharp materials. In addition to this, lack of monitoring of the standard infection prevention and control strategies may bring this horrible situation (See the revised version of the discussion in page 15 from lines 342-347.**

Reviewer #2: The authors have improved the paper very well. All the comments are properly addressed.
•The abstract is well structured,
•The background section is rearranged,
•The methodological issues are corrected,
•The write up of the results, discussion and conclusion section are improved,
•The implications of the study are indicated,
•Corrections are properly made on references,
•Writing and grammatical errors are corrected and
•Key notes of tables are properly defined and written.
By now, I will be happy if the paper is published at PLOS ONE.

**Response: Dear reviewers, we really appreciate your recognition for our manuscript. Thank you very much indeed.**
